# Body Shape and Weight Loss as Motivators for Breastfeeding Initiation and Continuation

**DOI:** 10.3390/ijerph14070754

**Published:** 2017-07-11

**Authors:** Sophie C. Schalla, Gemma L. Witcomb, Emma Haycraft

**Affiliations:** School of Sport, Exercise & Health Sciences, Loughborough University, Leicestershire LE11 3TU, UK; sophieschalla@gmail.com (S.C.S.); g.l.witcomb@lboro.ac.uk (G.L.W.)

**Keywords:** benefits, breastfeeding, breastfeeding initiation, breastfeeding continuation, promotion, psychological, body image, behaviour change

## Abstract

Breastfeeding rates in the UK are low. Efforts to promote breastfeeding typically include the known health benefits for mother and child, many of which are not immediate. Gaining immediate benefits can be effective motivators of behaviour. Body-related changes resulting from breastfeeding could be an immediate benefit. This study explored breastfeeding mothers’ reports of body-related changes as benefits of breastfeeding. Mothers (N = 182) who currently, or had recently, breastfed an infant completed a survey detailing their infant feeding choices and the perceived benefits of breastfeeding on their bodies. Half of the mothers felt that breastfeeding had a positive effect on their body. Benefits were grouped into five themes: (1) Returning to pre-pregnancy body shape; (2) Health benefits; (3) Physical benefits; (4) Eating benefits; (5) Psychological benefits. These themes highlight the numerous body-related benefits that mothers identified as resulting from breastfeeding and suggest that immediate, personal, and appearance-related gains of breastfeeding are highly valued. These findings indicate that interventions would likely benefit from emphasising the more immediate physical and psychological benefits of breastfeeding, alongside the health and bonding benefits, as a way to promote breastfeeding initiation and continuation in more women. This may be particularly effective for groups such as young mothers, where breastfeeding rates are low and whose emphasis on body image may be greater.

## 1. Introduction

The World Health Organization [1] recommends exclusive breastfeeding for the first six months of an infant’s life and continued breastfeeding until the infant reaches two years of age. While three-quarters of UK mothers (74% in 2014/2015) initiate breastfeeding, by 6–8 weeks post-birth fewer than half (47.2%) report exclusively breastfeeding their infants [2]. Therefore, it is clear that efforts are still required to better support mothers with breastfeeding initiation and maintenance. While guidelines recommend breastfeeding an infant up to two years of age, it is estimated that just 1% of mothers choose to do this [3].

Research suggests that there are many reasons mothers choose not to initiate breastfeeding. These may be related to perceptions surrounding the consequences of breastfeeding, such as pain and discomfort (e.g., [4]), a negative impact on breast shape [5], or feelings of embarrassment [6]. Moreover, of those mothers who do choose to initiate breastfeeding, many encounter problems which can lead to early cessation. Such factors include the perception that their milk supply is inadequate (e.g., [7]), and concern over the infant’s nutrition and lack of weight gain [8]. Physical pain can also lead to early cessation (e.g., [8]) as can emotional distress, such as depression or stress, and resultant feelings that breastfeeding is interfering with bonding. Furthermore, practical pressures, such as the management of other children and the feeling that breastfeeding takes up too much time [8], all play a part in why women may choose to cease breastfeeding.

To increase breastfeeding rates, women need to be motivated both to initiate and maintain breastfeeding and increasing breastfeeding relevant knowledge and skills through education is a crucial part of this [9]. There is a wealth of research attesting to the many health benefits for both mothers and their infants that result from breastfeeding (e.g., [10]) but despite mothers often having knowledge of these positive factors prenatally, many still choose to formula feed from birth. It is well established that intention is an important determinant of behaviour and Ajzen’s [11] Theory of Planned Behaviour has been shown to be highly relevant in the context of breastfeeding. Research has found that prenatal intention is predictive of actual infant feeding behaviour (e.g., [12]) and that positive breastfeeding attitudes predict breastfeeding intention [13]. Therefore, targeting feeding intentions prenatally would seem to be an effective way to promote breastfeeding. 

Research examining other health behaviours has found a positive association between perceived benefits and motivation (e.g., [14]) which suggests that promoting the positive benefits associated with breastfeeding might positively influence women who are yet to decide on their infant feeding method and continue to motivate mothers who are experiencing difficulties. Many of the benefits of breastfeeding that currently tend to be promoted are future, preventative benefits (e.g., protection from cancer). Arguably, breastfeeding initiation and maintenance rates may remain low because these messages do not emphasise the immediate gains and are therefore less effective in influencing underlying attitudes towards breastfeeding. Evidence suggests that people who engage in preventive health measures are those who have been able to see the benefit of making an initial investment in return for a long-term gain [15]. Therefore, interventions might be more successful if they are able to emphasise the benefits of breastfeeding that occur more immediately. Such benefits are likely to be psychological, rather than health-related.

One such benefit that is often praised by breastfeeding mothers, but is often underplayed in breastfeeding promotion literature, is that of weight loss and regaining their pre-pregnancy body shape. This may be because it is seen as an aesthetic benefit related to body image, and therefore something that should not be a main motivating factor. However, in western societies, where appearance and body image are of increasing importance to many women (and the “super-woman effect” exists, where mothers are under pressure to have careers, beauty, and raise children [16]), such messages may be motivating, particularly for young mothers. Data on the benefits of breastfeeding in relation to weight loss are mixed but four out of the five studies in Neville and colleagues’ [17] review that were deemed to be “of higher methodological quality in terms of weight measurements and adjustment for key covariates” (p. 587) found positive relationships between breastfeeding and weight loss. While basing breastfeeding interventions around aesthetics may seem undesirable, research suggests that interventions which offer valued rewards are likely to be successful, such as in the case of offering monetary rewards to encourage breastfeeding initiation [18].

However, the extent to which women value faster weight loss and see this as a legitimate and large enough gain given the heavy investment required to breastfeed is not known. The aim of this study was, therefore, to explore to what extent breastfeeding mothers report body-related changes as being a perceived benefit of breastfeeding. This information could be used in future breastfeeding promotion interventions to counteract negative beliefs about breastfeeding. 

## 2. Methods

### 2.1. Participants

Mothers (N = 233) with a child aged 7 to 24 months were recruited by opportunity sampling from playgroups, children’s centres or via online parenting forums and social networking sites. Those who reported not having breastfed were excluded, leaving a final sample of 182 mothers (mean age 32.68 years, standard deviation (SD) = 4.74, range 17 to 45). Most mothers (95.6%) identified themselves as White/Caucasian and their modal education level group was “University graduate” (40.7%). Mean child age was 15 months (SD = 5.10) with 51% of the children being male.

### 2.2. Measures and Procedure

Approval was granted by the Loughborough University Ethics Approvals (Human Participants) Sub-Committee for this cross-sectional study (G08-P1). Following this, consenting organisations (e.g., local playgroups, community groups, online parenting forums and social networking sites) were contacted and surveys were distributed to eligible mothers either on paper (face-to-face via the researcher) or via an online survey link. Surveys included an information sheet, consent form, demographic questions, and a series of questions developed to address this study’s aims, as outlined below.

Infant feeding choices: Mothers were asked about their infant feeding choices (“Is/was your child breastfed?”) with yes/no options. Mothers who reported breastfeeding were asked to state the duration of breastfeeding and of exclusive breastfeeding (i.e., when the child “received no other milk or solid food”). Mothers were also asked to indicate (yes/no) whether they were happy with their infant feeding decisions and also whether their infant feeding decisions were influenced by their beliefs regarding the effects it would have on their body (e.g., breasts, body shape, body size).

Perceived benefits of breastfeeding: Mothers were asked “Do you feel that breastfeeding has had any positive effects on your body?” with yes/no options given. Mothers who ticked ‘yes’ were then asked to provide details of the positive effects that they perceived and lines were provided for free-form written/typed responses.

### 2.3. Data Analysis

All quantitative data were entered into, and then analysed using, IBM SPSS Statistics 20 (IBM Corporation, Armonk, NY, USA). Missing data were omitted from relevant analyses. Descriptive statistics were calculated and then inductive thematic analysis, following the six steps outlined by Braun and Clarke [19], was used to assess the reported benefits of breastfeeding. One researcher (Sophie C. Schalla) conducted the initial thematic analysis and 20% was checked and verified by a second researcher (Emma Haycraft). This method of assessment of the validity of coding has been widely used in previous research and is acknowledged as appropriate for such a thematic analysis [20].

## 3. Results

### 3.1. Descriptive Statistics

The median breastfeeding duration was 6.00 months (IQR 6.00; mean 6.41 months; SD = 4.15) while the median duration of exclusive breastfeeding was 5.00 months (IQR 3.50; mean 4.24 months; SD = 2.39). The majority of mothers (89%, N = 161) indicated that they were happy with the decisions they had made surrounding their infant’s feeding. While only 14% (N = 24) of mothers reported that their feeding decisions were influenced by their beliefs about the positive effects that breastfeeding might have on their body, half of the mothers (51%, N = 91) indicated that they felt breastfeeding had a positive effect on their body. Benefits of breastfeeding were further explored via mothers’ free-form responses and analysed via inductive thematic analysis.

### 3.2. Inductive Thematic Analysis

Following familiarisation with the data and initial generation of codes, a series of themes were produced. These themes were reviewed, finalised and named. The five themes can be seen in Table 1. 

Theme 1 refers to how breastfeeding has helped mothers to return to their pre-pregnancy body shape, and includes positive physical changes that the mothers attribute to breastfeeding. Theme 2 addresses the short- and long-term health benefits of breastfeeding. Theme 3 focuses on the physical benefits which mothers reported that they considered breastfeeding to contribute to (excluding weight-loss or uterine contraction). Theme 4 reflects many mothers’ reports that breastfeeding had positively affected their diet and eating habits. Finally, Theme 5 represents the psychological benefits mothers reported having gained from breastfeeding.

## 4. Discussion

This study aimed to examine the benefits of breastfeeding and the extent to which weight loss/body changes are regarded as positive and/or unanticipated benefits, as reported by mothers who are currently breastfeeding or have recently breastfed. A variety of physical and psychological benefits of breastfeeding were identified. The data indicated that weight loss, and regaining pre-pregnancy body shape, was the most cited positive consequence of breastfeeding (reported by 95% of our sample), supporting previous research (e.g., [21]). However, only 14% of women reported that their decision to breastfeed was influenced by this knowledge. This suggests that breastfeeding information and interventions may not be doing enough to highlight the positive effects on post-pregnancy body weight that breastfeeding can have.

Mothers’ comments highlighted that weight loss, weight loss in comparison to other mothers, and the ability to eat a lot whilst either maintaining a slim body or not gaining weight were all important benefits that they gained by breastfeeding and which they felt would help them regain their pre-pregnancy body shape. This suggests that immediate, personal, and appearance-related gains of breastfeeding are given high value by mothers. While caution would need to be taken when promoting weight loss as a reason to breastfeed due to the implications for encouraging over-evaluation of the self in terms of body shape and weight (a risk factor for the development of eating disorders [22]), these data do provide valuable insight into what the motivating factors are that may help women to initiate and maintain breastfeeding.

As well as weight loss, other benefits to the body were identified, such as having good skin, good hair, and hormone regulation. Future physical benefits were also mentioned, including reduced risks of breast and ovarian cancers and type 2 diabetes, supporting previous literature in the area (e.g., [23]). Notably, in addition, despite being asked about their bodies, mothers also reported perceived breastfeeding benefits that were psychological. Not only did they describe mood benefits, supporting previous research which suggests that breastfeeding may be linked to reductions in negative maternal mood [24], but they also reported enhanced self-esteem, feeling proud of being able to breastfeed, and feeling valued and with purpose. These potential psychological benefits of breastfeeding have been less well documented in the literature to date. However, self-efficacy is increasingly being seen as a crucial marker for success and resilience [25] and may therefore be important in helping women decide whether to initiate breastfeeding or to help navigate problems with breastfeeding if/when they occur. Indeed, while improved mood may be related to hormonal changes associated with lactation, recent research suggests that bolstering women’s breastfeeding self-efficacy in the early post-partum days may also be crucial for enhancing post-birth mood and prolonging breastfeeding duration [26].

Our study highlights that mothers who have recently breastfed/are currently breastfeeding regard weight loss and aesthetic appearance gains as a major benefit of breastfeeding. However, it should be noted that although the study included a large sample size with diverse breastfeeding durations, the sample was predominantly white British and so these factors may not be as important in culturally diverse samples who may not regard weight loss and body shape as being as important. It is noted that data were retrospective for those mothers who had ceased breastfeeding and this might have created a degree of recall bias. Our definition of exclusive breastfeeding differed slightly from that of the World Health Organization as it did not distinguish between “other milk” and “other drink”—i.e., water—and this limitation needs to be acknowledged. Moreover, as some participating mothers were still breastfeeding information was not collected from everyone on overall breastfeeding duration, or on intensity of breastfeeding; both of which would likely be important in framing and tempering any proposed health promotion campaigns related to weight loss and breastfeeding, and so future research should collect this information.

## 5. Conclusions

Overall, the results of this novel study suggest that interventions aimed at increasing breastfeeding initiation and maintenance rates should highlight the array of benefits that breastfeeding can bring, with a particular focus on the immediate gains and those that may be relevant for increasing self-esteem. These benefits appear to be broader and more immediate than protection against later health problems. Given the evidence which suggests that gaining more immediate benefits can be a particularly effective motivator of behaviour for some individuals [15], further interventions could be effectively developed which emphasise the physical and psychological benefits of breastfeeding, in addition to health and bonding benefits, in an effort to promote breastfeeding initiation and continuation in more women. This may be particularly effective for specialist groups, such as young mothers, where breastfeeding rates are low and who may place greater emphasis on body image.

## Figures and Tables

**Table 1:** Summary of the themes identified via inductive thematic analysis relating to the perceived benefits of breastfeeding identified by mothers.

Theme	Subtheme	Example Quotation (Participant ID)
1. Returning to pre-pregnancy body shape	Loss of baby weight	“I've lost 2.5 stone in 7 months and I think that’s partly due to breastfeeding” (M 11) “I lost the baby weight quicker than friends who didn’t breastfeed” (M 43)
	Uterus contraction/flatter stomach	“It helped my uterus contract faster” (M 7) “Uterus shrunk back quicker” (M 44)
		“Tummy reduced quickly after birth” (M 4) “Helped stomach return to almost normal quicker” (M 47)
2. Health benefits (for mother and/or child)		“Reduces cancer risks, reduces weight, reduces diabetes and other health benefits to baby” (M 6)
3. Physical benefits		“Immediately, better hormonal regulation” (M 48) “My skin and hair still look great” (M 18)
4. Eating benefits		“I was able to eat more food without gaining weight” (M 24) “I’m smaller than pre-pregnancy and I eat like a horse!” (M 28) “I also ate healthier than I normally would as I was aware that I was feeding my baby” (M 61) “I can eat more because I burn more calories” (M 67)
5. Psychological benefits		“I feel comfortable in my own skin!” (M 8) “Proud my body was able to do it” (M 47) “Made me a more confident, less shy person” (M 52) “Made me feel more comfortable with my body” (M 62) “Mood more balanced” (M 68) “It made it feel useful” (M 88)
